# Research on Adaptive Variable Impedance Control Method Based on Adaptive Neuro-Fuzzy Inference System

**DOI:** 10.3390/s25103055

**Published:** 2025-05-12

**Authors:** Xianlun Wang, Chuanhuan Li, Dexin Cai, Yuxia Cui

**Affiliations:** College of Electromechanical Engineering, Qingdao University of Science and Technology, Qingdao 266000, China; xlwang@126.com (X.W.);

**Keywords:** manipulator, nonlinear tracking differentiator, adaptive variable impedance control, adaptive neuro-fuzzy inference system

## Abstract

Precise force tracking and overshoot suppression are critical for manipulator dynamic contact tasks, especially in unstructured environments such as complex surface cleaning that rely on dynamic feedback from force sensors. Traditional impedance control methods exhibit limitations through excessive force overshoot and steady-state error, severely impacting cleaning performance. To address this problem, this paper introduces proportional–integral–derivative (PID) control based on the traditional impedance model and verifies the stability and convergence of the controller through theoretical analysis. Meanwhile, to improve the applicability of the controller and avoid using expert experience to formulate fuzzy rules, this paper designs an adaptive neuro-fuzzy inference system (ANFIS) to dynamically adjust the update rate. To validate the effectiveness of the proposed method, simulation experiments mirroring real-world scenarios of contact cleaning tasks are constructed in Simulink. The results demonstrate that, compared to adaptive impedance control (AIC) and adaptive variable impedance control (AVIC), the proposed controller achieves a faster steady-state response and exhibits negligible overshoot and minimal force steady-state error during both constant and sinusoidal force tracking. Furthermore, the controller demonstrates superior stability under abrupt changes in stiffness and desired force.

## 1. Introduction

The rapid evolution of robotics and sensor technology has expanded manipulator applications across the industrial, medical, and service domains. For example, in industrial manufacturing, manipulators are widely used for polishing, grinding, and welding of parts [1,2,3] and to improve the quality of machined surfaces by embedding force/torque sensors; in the medical field, manipulators assist doctors in medical rehabilitation by compensating for hand tremors to avoid damage to surrounding tissues [4,5]; and, within domestic service scenarios, manipulators can execute cleaning tasks, such as wiping tables and windows [6], and regulate the cleaning strength through force sensor feedback. In these applications, manipulators are sometimes required to physically interact with complex environments and, at other times, share workspaces with humans [7]. Crucially, the end-effector of the manipulator needs to track the position while applying a certain force to the external environment based on the feedback from the sensors. Consequently, the design of safer and more efficient compliant control strategies is not only a central focus in the field of manipulator control, but also drives the sensor technology in the direction of high response.

Compliant control strategies for the manipulator include active and passive compliance control [8]. Active compliance control includes hybrid force/position control (HFPC) and impedance control [9]. Conventional impedance control employs fixed-parameter models where pre-defined stiffness, damping, and inertia dictate environmental interactions. These parameters are typically pre-set before task execution, based on task requirements, and depend on an accurate environmental model. While effective in structured settings, this static approach fails in unstructured environments due to dynamic uncertainties and the absence of real-time impedance adaptation [10]. And the moment the end-effector of the manipulator comes into contact with the environment, it generates a large force overshoot. Methods must be employed to reduce this overshoot to ensure better system stability [11].

Addressing the limitations of conventional impedance control, prevalent solutions include the following: (1) direct reference trajectory adjustment: force tracking is achieved by directly modifying the reference trajectory based on feedback information [12]; (2) indirect reference trajectory adjustment: this approach primarily utilizes environmental identification to ascertain environmental stiffness and position, thereby indirectly deriving the desired reference trajectory for force tracking [13,14]; and (3) adaptive impedance control: this type of method combines an adaptive mechanism on the impedance model, which is mainly combined with fuzzy control [15,16,17], neural networks [18,19], and adaptive control strategies [20,21,22] to dynamically adjust the impedance parameters according to the force error and realize the tracking of the force. For instance, WANG [23] proposed a PD adaptive impedance control method, which effectively reduced force-tracking errors and force overshoot. GU [24] introduced a fractional-order PID adaptive impedance control strategy that can dynamically assess environmental information and update the reference trajectory in unstructured environments, effectively reducing force-tracking errors. SONG [25] employed an adaptive control strategy to adaptively adjust the damping and stiffness coefficients in impedance control and experimentally validated that this method does not exhibit contact force overshoot. CHEN [26] designed an adaptive impedance control algorithm that utilizes an adaptive compensation controller to effectively mitigate contact forces during impact. CAO proposed adaptive hybrid impedance control strategies [27] and smooth adaptive hybrid impedance control strategies [28] for the manipulator contact force-tracking tasks in dynamic environments, demonstrating favorable force-tracking performance. To achieve compliant control of the end-effectors in a dual-manipulator system, LI [29] designed an impedance control method based on a particle swarm optimization algorithm, which reduces overshoot while maintaining good stability. KONG [30] proposes a variable universe fuzzy model reference adaptive impedance control method, which employs a variable universe fuzzy controller to adjust the impedance parameters and achieves good control results in simulation. Pankert [31] actively adapts the target trajectory to the contact force requirements by generating position and attitude corrections via an admittance controller, which are then fed into the MPC for optimization. In this way, the MPC considers both trajectory tracking and force control goals when planning future actions, but requires an accurate model of the robot and environment interaction. Ding [32] dynamically optimizes the impedance control parameters of the manipulator through reinforcement learning algorithms, establishes a Gaussian process model to predict the state transfer, and searches for the optimal parameters using a gradient strategy, so that the manipulator can stably track the target contact force during the polishing process. However, reinforcement learning algorithms are less generalizable and cannot be directly adapted to other platforms.

To address the force overshoot issue during contact, nonlinear feedback control methods can be employed to reduce the overshoot magnitude [33]. While ROVEDA [34] utilizes impedance shaping to mitigate force overshoot, its practical application is limited by the requirement for precise estimation of environmental position and stiffness. WU [33] and XIE [35] adopted a nonlinear tracking differentiator (NTD) to smooth step-force transitions, and validated the effectiveness of this method in grinding and polishing scenarios.

This paper introduces AVIC for cleaning tasks. The challenge lies in the unevenness of the cleaning surface, requiring the manipulator to adapt to different surface shapes based on force sensor feedback while applying the appropriate force to complete the cleaning task. Addressing the limitations of traditional impedance control in achieving effective force tracking for cleaning tasks, we propose an ANFIS-based adaptive variable impedance controller. The controller synergistically combines the NTD and ANFIS. Initially, the nonlinear tracking differentiator smooths the desired force to reduce force overshoot. Subsequently, an AVIC framework is designed, which introduces PID control based on the conventional impedance control, proportional (P) control at the update rate, and adaptive tuning of damping coefficients and stiffness coefficients through the adaptive control law. Finally, to address the problem that traditional fuzzy control needs to rely on experts’ experience, ANFIS is introduced to output smoother fuzzy rules to adjust the update rate in real time.

The paper is organized as follows: Section 2 details the construction of the dynamic contact force model, incorporating an NTD approach for smoothing the desired force profile. In Section 3, the design of an adaptive variable impedance controller is first presented and the stability and convergence conditions of the algorithm are demonstrated. ANFIS was then designed to adaptively adjust the update rate through its neuro-fuzzy architecture. In Section 4, to validate the effectiveness of the proposed method in this paper, simulations are performed in a MATLAB/Simulink environment. Finally, in Section 5, the content of the article is summarized.

## 2. System Model and Force Overshoot Suppression Strategy

**Figure 1:**
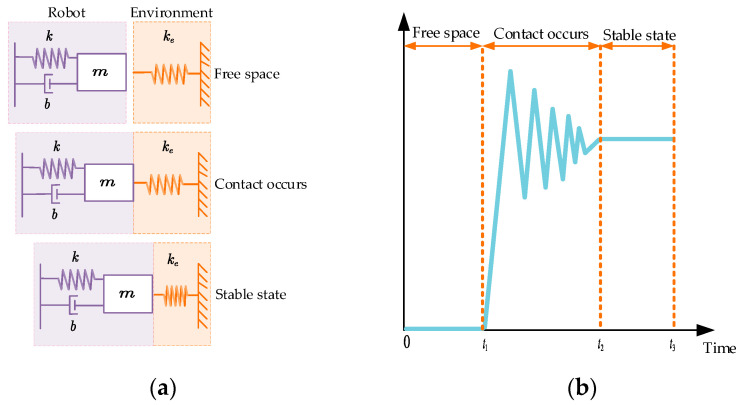
Contact process between the end-effector of the manipulator and task environment: (**a**) contact model; and (**b**) visualization of dynamic contact force variation during interaction processes.

To accurately characterize the dynamic relationship between the end-effector of the manipulator and environmental contact forces, it is essential that we establish a corresponding contact dynamics model. Assuming that the environment is rigid, reference [22] proposes the theory of position impedance control, which usually reduces the environment to a first-order spring system. The contact dynamics model is shown in Figure 1a. The contact process evolves from free-space motion to the critical contact point, followed by gradual force stabilization. During this process, the manipulator’s contact forces undergo continuous variations, as depicted in Figure 1b, which can be systematically divided into three distinct phases:

From 0 to $t_{1}$: The manipulator approaches the operational environment without physical contact.

From $t_{1}$ to $t_{2}$: Transient collision occurs upon initial contact between the end-effector and the environment, during which contact forces exhibit pronounced fluctuations.

From $t_{2}$ to $t_{3}$: Post-contact force progressively stabilizes to equilibrium.

In the case where the environment information is unknown, the initial environment position is usually used to replace the desired position, and the force/torque of the manipulator in Cartesian space is decoupled [22]. In this paper, the force on the Z-axis is taken as the research object, and the dynamic contact force model of the manipulator with the environment is shown in Equation (1):

(1) $$m(\ddot{x}-{\ddot{x}}_{e})+b(\dot{x}-{\dot{x}}_{e})+k(x-x_{e})=f_{d}-f_{e}=\Delta f$$

where m, b, k are the mass, damping, and stiffness coefficients of the end-effector, $f_{d}$ is the desired contact force, $f_{e}$ is the actual contact force as measured by the force transducer, and Δf is the force-tracking error. The model establishes the dynamic mapping between the end displacement x and the contact force through the physical coupling relationship of the mass–spring–damping system.

When impedance control is used for force tracking, the real-time feedback results from the force sensor are shown in Figure 1b. It can be seen that the transition from the uncontacted state to the contacted state of the manipulator produces a short collision process, resulting in a large force overshoot at the moment of contact with the environment. To solve this problem, in this paper, a discrete form of NTD [36] is used to smooth the transition process of $f_{d}$. The discrete form of NTD dynamically adjusts the state update by designing a nonlinear function to construct a control strategy using the error and differential signals, combined with a filtering factor h, a sampling rate T, and a fast factor r. The difference equation in this paper contains two states $v_{1}$ and $v_{2}$, representing the smoothed force and force differential signals, respectively, and is implemented using the fhan function:

(2) $$\left\{\begin{array}{l} \mathrm{fh}=\mathrm{fhan}(v_{1}(k)-f_{d}(t),v_{2}(k),r,h)\\ v_{1}(k+1)=v_{1}(k)+Tv_{2}(k)\\ v_{2}(k+1)=v_{2}(k)+T\mathrm{fh} \end{array}\right.$$

The $f_{0}=fhan(x_{1},x_{2},r,h)$ function in Equation (2) above is as follows [36]:

(3) $$\left\{\begin{array}{l} d=rh^{2}\\ a_{0}=hx_{2}\\ y=x_{1}+a_{0}\\ a_{1}=\sqrt{d(d+8|y|})\\ a_{2}=a_{0}+sign(y)(a_{1}-d)/2\\ a=(a_{0}+y)fsg(y,d)+a_{2}(1-fsg(y,d))\\ \;fhan=-r\left(\frac{a}{d}\right)fsg(a,d)-rsign(a)(1-fsg(a,d)) \end{array}\right.$$

In the above Equation (3), fsg(x,d)=(sign(x+d)−sign(x−d))/2. Subsequently, the control mode is switched based on the function fsg(x,d): when the prediction error $\left|y\right|>d$, the linear mode $a=(a_{0}+y)/h$ is used to quickly approximate the target signal, and, vice versa, $a=a_{2}$ is maintained to suppress the noise. Eventually, the reverse acceleration is dynamically adjusted by the nonlinear input $fhan=-r\left(\frac{a}{d}\right)fsg(a,d)-rsign(a)(1-fsg(a,d))$ to avoid force overshooting while ensuring the tracking velocity.

**Figure 2:**
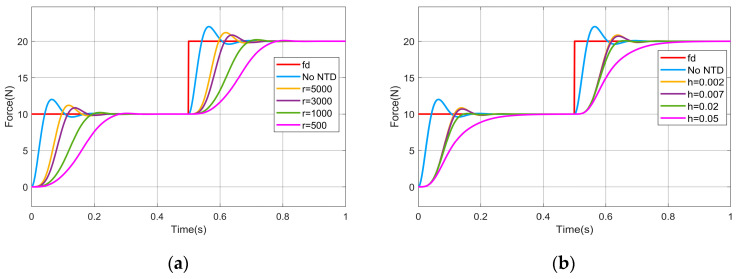
Output of optimization of impedance control using NTD: (**a**) use different values of r; (**b**) use different values of h.

In order to demonstrate that the NTD smoothing of $f_{d}$ can effectively reduce the amount of force overshoot, the NTD is added to the impedance control module for simulation experiments, and the Transfer Fcn module in MATLAB 2023b/Simulink is used to simulate the impedance controller, and the parameters of the simulation process are set as follows: the sampling rate T=0.001 s, the expected force $f_{d}$ in the step is 10 N in the first 0.5 s, and 20 N in the second 0.5 s, the inertia coefficient m=1, the damping coefficient b=50, the stiffness coefficient k=0, the environmental stiffness k=3000, and the simulation time is 1 s. The simulation results are shown in Figure 2.

From Figure 2, it can be seen that the maximum overshoot of the contact force is about 2 N when impedance control is used, while the maximum overshoot of the contact force will be reduced when the $f_{d}$ is smoothed using NTD. Specifically, when the value of the filtering factor h is fixed (h=0.002), the smaller the value of the rapidity factor r is, and the smaller the maximum overshoot of the contact force is; and, when the rapidity factor r=500, the maximum overshoot of the contact force is only 0.1 N. When the value of the rapidity factor r is fixed (r=3000), the larger the value of the filtering factor h is, and the smaller the maximum overshoot of the contact force is, and, when the filtering factor h=0.05, there is no contact force overshoot.

## 3. Design of an Adaptive Variable Impedance Controller Based on ANFIS

In the process of performing the cleaning task, the cleaning surface may be a more complex curved surface, and the prerequisite of the multi-variable curvature requires that the end-effector of the manipulator maintains a suitable contact force and relative position with the cleaning surface. In accordance with the specific requirements of the mandate, this section investigates the position-based impedance control model and combines it with the adaptive control strategy and ANFIS to design an adaptive variable impedance controller based on ANFIS, which contains the following: (1) an adaptive variable impedance controller is designed, which can adaptively adjust the damping and stiffness coefficients; (2) the stability and convergence of the adaptive variable impedance control algorithm were analyzed, and the stability boundary conditions of the algorithm were obtained; and (3) ANFIS was used to adaptively adjust the update rate instead of the traditional fuzzy controller.

### 3.1. Design of an Adaptive Variable Impedance Controller

When a manipulator performs a cleaning task, the operating environment usually has non-structural characteristics, such as more complex curved surfaces. In this kind of environment position, the traditional impedance control cannot realize better force tracking due to the dependence on fixed environment parameters, and, thus, cannot complete the work of cleaning tasks, which requires the estimation of the position of the environment. Assuming the environment prediction value ${\hat{x}}_{e}=x_{e}+\eta x_{e}$, η is the environment position perturbation; at this time, the corresponding trajectory error is $\hat{e}=e_{r}-\eta x_{e}$, which is brought into Equation (1) to obtain the force-tracking error Δf≠0.

To eliminate the steady-state error in force tracking, AVIC can be used to adjust the impedance parameters to adapt to the environmental changes, but, to prevent the system from oscillating, we generally do not modify the mass parameters [33]. Therefore, in this paper, the damping coefficients and stiffness coefficients are dynamically adapted through the use of adaptive control law. Meanwhile, to improve the response speed of the system and reduce the force overshoot, PID control is introduced based on traditional impedance control, and proportional P control is introduced at the update rate, by which the adaptive variable impedance control (PID-P-AVIC) equation obtained is as follows:

(4) $$m\ddot{\hat{e}}(t)+(b+\Delta b(t))\dot{\hat{e}}(t)+(k+\Delta k(t))\hat{e}(t)=k_{p}\Delta f+k_{i}\int_{0}^{\tau }\Delta fd\tau +k_{d}\Delta \dot{f}$$

where $k_{p},\;k_{i},\;k_{d}$ are the proportional, integral, and differential coefficients in PID control. The expressions for the damping compensation coefficient Δb(t) and the stiffness compensation coefficient Δk(t) are shown in Equation (5):

(5) $$\left\{\begin{array}{c} \Delta b(t)=\frac{b}{\dot{\hat{e}}(t)+\lambda }\Gamma_{b}(t)\\ \Gamma_{b}(t)=\Gamma_{b}(t-T)-\sigma \frac{k_{p}\Delta f(t-T)}{b}\\ \Delta k(t)=\frac{k}{\hat{e}(t)+\lambda }\Gamma_{k}(t)\\ \Gamma_{k}(t)=\Gamma_{k}(t-T)-\sigma \frac{k_{p}\Delta f(t-T)}{k} \end{array}\right.$$

**Figure 3:**
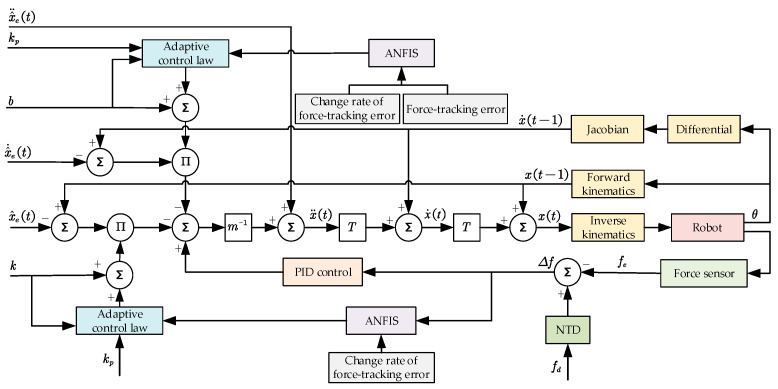
Principle of adaptive variable impedance controller based on ANFIS.

In the above Equation (5), Γ(t) is the amount of adaptive compensation at period t. T is the sampling rate. σ is the update rate, which is used to represent the adaptive gain of the variable. To prevent the denominator in the above equation from being 0, $\lambda =10^{-8}$ is set. The final obtained AVIC principle based on ANFIS is shown in Figure 3.

### 3.2. Stability and Convergence Analysis of Adaptive Variable Impedance Controllers

In this section, we will give the constraints when the AVIC of this paper is stabilized and prove that the steady-state error of force tracking is 0, based on the stability and convergence proof methods in the literature [25,28]. Firstly, Equation (4) is expanded as follows:

(6) $$m\ddot{\hat{e}}(t)+b\dot{\hat{e}}(t)+k\hat{e}(t)+b\Gamma_{b}(t-T)+k\Gamma_{k}(t-T)-2\sigma k_{p}\Delta f(t-T)=k_{p}\Delta f+k_{i}\int_{0}^{\tau }\Delta fd\tau +k_{d}\Delta \dot{f}$$

Bringing the trajectory error $\hat{e}=e_{r}-\eta x_{e}$ into Equation (6) gives the following:

(7) $$\begin{array}{c} m({\ddot{e}}_{r}(t)-\eta {\ddot{x}}_{e}(t))+b({\dot{e}}_{r}(t)-\eta {\dot{x}}_{e}(t))+k(e_{r}(t)-\eta x_{e}(t))+b\Gamma_{b}(t-T)+k\Gamma_{k}(t-T)-2\sigma k_{p}\Delta f(t-T)\\ =k_{p}\Delta f+k_{i}\int_{0}^{\tau }\Delta fd\tau +k_{d}\Delta \dot{f} \end{array}$$

Organizing Equation (7) gives the following:

(8) $$\begin{array}{c} m{\ddot{e}}_{r}(t)+b{\dot{e}}_{r}(t)+ke_{r}(t)+b\Gamma_{b}(t-T)+k\Gamma_{k}(t-T)-2\sigma k_{p}\Delta f(t-T)-(k_{p}\Delta f+k_{i}\int_{0}^{\tau }\Delta fd\tau +k_{d}\Delta \dot{f})\\ =m\eta {\ddot{x}}_{e}(t)+b\eta {\dot{x}}_{e}(t)+k\eta x_{e}(t) \end{array}$$

Since the environment is assumed to be a first-order spring system, the contact force between the robot and the environment can be simplified to $f_{e}=k_{e}(x-x_{e})=k_{e}e_{r}$, we obtain $e_{r}=f_{e}/k_{e}$, ${\dot{e}}_{r}={\dot{f}}_{e}/k_{e}$, ${\ddot{e}}_{r}={\ddot{f}}_{e}/k_{e}$, which can be obtained by taking it into Equation (8):

(9) $$\begin{array}{c} m{\ddot{f}}_{e}(t)+b{\dot{f}}_{e}(t)+kf_{e}(t)+bk_{e}\Gamma_{b}(t-T)+kk_{e}\Gamma_{k}(t-T)-2\sigma k_{e}k_{p}\Delta f(t-T)-k_{e}(k_{p}\Delta f+k_{i}\int_{0}^{\tau }\Delta fd\tau +k_{d}\Delta \dot{f})\\ =mk_{e}\eta {\ddot{x}}_{e}(t)+bk_{e}\eta {\dot{x}}_{e}(t)+kk_{e}\eta x_{e}(t) \end{array}$$

Setting the environmental estimated force error ${\hat{f}}_{e}(t)=k_{e}\eta x_{e}(t)$, Equation (9) is expressible in a simplified form:

(10) $$\begin{array}{c} m{\ddot{f}}_{e}(t)+b{\dot{f}}_{e}(t)+kf_{e}(t)+bk_{e}\Gamma_{b}(t-T)+kk_{e}\Gamma_{k}(t-T)-2\sigma k_{e}k_{p}\Delta f(t-T)-k_{e}(k_{p}\Delta f+k_{i}\int_{0}^{\tau }\Delta fd\tau +k_{d}\Delta \dot{f})\\ =m{\ddot{\hat{f}}}_{e}(t)+b{\dot{\hat{f}}}_{e}(t)+k{\hat{f}}_{e}(t) \end{array}$$

Equation (10) can be simplified by adding both sides of Equation (10) simultaneously with the equation $-m{\ddot{f}}_{d}(t)-b{\dot{f}}_{d}(t)-kf_{d}(t)$, and, in order to simplify the subsequent analysis, new variables $\psi (t)=f_{e}(t)-f_{d}(t)$ and $\phi (t)={\hat{f}}_{e}(t)-f_{d}(t)$ are introduced, and Equation (10) can be simplified to:

(11) $$\begin{array}{c} \;\;m\ddot{\phi }(t)+b\dot{\phi }(t)+k\phi (t)\\ =m\ddot{\psi }(t)+b\dot{\psi }(t)+k\psi (t)+k_{e}k_{p}\psi (t)+k_{e}k_{i}\int_{0}^{\tau }\psi (t)d\tau +k_{e}k_{d}\dot{\psi }(t)+bk_{e}\Gamma_{b}(t-T)+kk_{e}\Gamma_{k}(t-T)+2\sigma k_{e}k_{p}\psi (t-T) \end{array}$$

For the adaptive compensation quantity $\Gamma_{b}(t-T)$ in the period (t−T), it satisfies the following:

(12) $$\Gamma_{b}(t-T)=\Gamma_{b}(t-2T)+\frac{\sigma k_{p}}{b}\psi (t-2T)$$

Similarly, the compensation terms for preceding sampling periods (t−2T),(t−3T),…,(t−nT) can be derived as $\Gamma_{b}(t-2T),\Gamma_{b}(t-3T),\ldots ,\Gamma_{b}(t-nT)$, as formalized in Equation (13):

(13) $$\begin{array}{c} \Gamma_{b}(t-2T)=\Gamma_{b}(t-3T)+\frac{\sigma k_{p}}{b}\psi (t-3T)\\ \Gamma_{b}(t-3T)=\Gamma_{b}(t-4T)+\frac{\sigma k_{p}}{b}\psi (t-4T)\\ \vdots \\ \Gamma_{b}(t-nT)=\Gamma_{b}(t-(n+1)T)+\frac{\sigma k_{p}}{b}\psi (t-(n+1)T) \end{array}$$

The equations in Equation (13) can be obtained by adding up the equations:

(14) $$bk_{e}\Gamma_{b}(t-T)=bk_{e}\Gamma_{b}(t-(n+1)T)+\sigma k_{e}k_{p}(\psi (t-(n+1)T)+\cdots +\psi (t-2T))$$

Since the initial value of Γ is generally set to 0, i.e., $\Gamma_{b}(t-(n+1)T)=0$, this is obtained by taking it into Equation (14):

(15) $$bk_{e}\Gamma_{b}(t-T)=\sigma k_{e}k_{p}(\psi (t-(n+1)T)+\cdots +\psi (t-2T))$$

Equation (16) can be obtained by the same reasoning:

(16) $$kk_{e}\Gamma_{k}(t-T)=\sigma k_{e}k_{p}(\psi (t-(n+1)T)+\cdots +\psi (t-2T))$$

Equation (11) is organized as follows:

(17) $$\begin{array}{c} m\ddot{\phi }(t)+b\dot{\phi }(t)+k\phi (t)\\ =m\ddot{\psi }(t)+b\dot{\psi }(t)+k\psi (t)+k_{e}k_{p}\psi (t)+k_{e}k_{d}\dot{\psi }(t)+k_{e}k_{i}\int_{0}^{\tau }\psi (t)d\tau +2\sigma k_{e}k_{p}(\psi (t-(n+1)T)+\cdots +\psi (t-T)) \end{array}$$

Performing Laplace transformation on Equation (17) yields the following:

(18) $$\frac{\psi (s)}{\phi (s)}=\frac{ms^{2}+bs+k}{ms^{2}+(b+k_{e}k_{d})s+k_{e}k_{p}+k+k_{e}k_{i}\frac{1}{s}+2\sigma k_{e}k_{p}(e^{-(n+1)Ts}+\cdots +e^{-Ts})}$$

The characteristic equation derived from Equation (18) is expressed as follows:

(19) $$ms^{2}+(b+k_{e}k_{d})s+k_{e}k_{p}+k+k_{e}k_{i}\frac{1}{s}+2\sigma k_{e}k_{p}(e^{-(n+1)Ts}+\cdots +e^{-Ts})=0$$

Multiply the left and right sides of Equation (19) by s at the same time to obtain the following:

(20) $$ms^{3}+(b+k_{e}k_{d})s^{2}+(k_{e}k_{p}+k)s+k_{e}k_{i}+2s\sigma k_{e}k_{p}(e^{-(n+1)Ts}+\cdots +e^{-Ts})=0$$

For sufficiently large n, the following asymptotic relation holds:

(21) $$\sum_{n=1}^{\infty }e^{-nTs}=\frac{e^{-Ts}}{1-e^{-Ts}}$$

The sampling period T is assumed to be small enough to ensure that the discrete-time model closely approximates the continuous-time dynamic properties. At this point, the Taylor expansion equation shows that $e^{-Ts}\approx 1-Ts$. Substituting this approximation into Equation (20) yields the following:

(22) $$ms^{3}+(b+k_{e}k_{d})s^{2}+(k_{e}k_{p}+k)s+k_{e}k_{i}+2s\sigma k_{e}k_{p}\frac{1-Ts}{Ts}=0$$

Equation (22) is obtained by organizing the following:

(23) $$Tms^{3}+(b+k_{e}k_{d})Ts^{2}+(k_{e}k_{p}+k-2\sigma k_{e}k_{p})Ts+Tk_{e}k_{i}+2\sigma k_{e}k_{p}=0$$

Following the Routh–Hurwitz stability criterion, the Routh array is systematically constructed as presented below:
$s^{3}$Tm$(k_{e}k_{p}+k-2\sigma k_{e}k_{p})T$$s^{2}$$(b+k_{e}k_{d})T$$Tk_{e}k_{i}+2\sigma k_{e}k_{p}$$s^{1}$$\frac{T^{2}(b+k_{e}k_{d})(k_{e}k_{p}+k-2\sigma k_{e}k_{p})-Tm(Tk_{e}k_{i}+2\sigma k_{e}k_{p})}{(b+k_{e}k_{d})T}$0$s^{0}$$Tk_{e}k_{i}+2\sigma k_{e}k_{p}$0

To ensure the stability of the control system, the Routh–Hurwitz stability conditions mandate that all first-column elements in the Routh array and all coefficients of the characteristic equation must satisfy strict positivity constraints, i.e., the following:

(24) $$\left\{\begin{array}{l} \begin{array}{c} \frac{T^{2}(b+k_{e}k_{d})(k_{e}k_{p}+k-2\sigma k_{e}k_{p})-Tm(Tk_{e}k_{i}+2\sigma k_{e}k_{p})}{(b+k_{e}k_{d})T}>0\\ (k_{e}k_{p}+k-2\sigma k_{e}k_{p})T>0\\ Tk_{e}k_{i}+2\sigma k_{e}k_{p}>0 \end{array} \end{array}\right.$$

Solving the aforementioned inequalities yields the stability conditions for the adaptive variable impedance controller as follows:

(25) $$\left\{\begin{array}{c} \begin{array}{c} \frac{-k_{i}T}{2k_{p}}<\sigma <\frac{(Tb+Tk_{e}k_{d})(k_{e}k_{p}+k)-mTk_{e}k_{i}}{2k_{e}k_{p}(m+Tb+Tk_{e}k_{d})}\\ \frac{-k_{i}T}{2k_{p}}<\sigma <\frac{k_{e}k_{p}+k}{2k_{e}k_{p}}=0.5+\frac{k}{2k_{e}k_{p}} \end{array} \end{array}\right.$$

For a stable system, the force steady-state error $e_{ss}$ can be derived from its Laplace-domain representation. The error calculation methodology is formalized in Equation (26):

(26) $$\begin{array}{c} e_{ss}=\underset{s\to 0}{\lim}sE(s)=\underset{s\to 0}{\lim}s(\psi (s)-\phi (s))\\ =\underset{s\to 0}{\lim}\left[\frac{ms^{2}+bs+k}{ms^{2}+(b+k_{e}k_{d})s+k_{e}k_{p}+k+k_{e}k_{i}s^{-1}+2\sigma k_{e}k_{p}(e^{-(n+1)Ts}+\cdots +e^{-Ts})}-1\right]\phi (s) \end{array}$$

where E(s) denotes the error signal in the Laplace domain. When the system is subjected to a unit step input ϕ(s)=1/s, the force steady-state error $e_{ss}$ is computed as follows:

(27) $$e_{ss}=\underset{s\to 0}{\lim}s(\psi (s)-\phi (s))=-1$$

Thus, the conclusive result is derived as follows:

(28) $$\underset{s\to 0}{\lim}s\psi (s)=0,\underset{t\to \infty }{\lim}\psi (t)=0$$

The analytical derivations conclusively demonstrate that the actual contact force $f_{e}$ asymptotically converges to the desired profile $f_{e}$ at steady state t→∞. Notably, the proposed control architecture maintains robust tracking precision even under time-varying reference signals with complex spectral components.

To ensure the successful execution of simulation experiments, systematic parameter selection is conducted under the following principles: The proportional gain $k_{p}$ is typically set to be greater than 1 to optimize transient response dynamics. For the integral gain $k_{i}$, it should be ensured that its effect on the lower limit of the update rate is small; ideally, the lower limit of the update rate should be close to 0, which helps in the subsequent ANFIS design. Therefore, $k_{i}$ should be selected to be as small as possible while maintaining controller stability. To mitigate the impact of environmental stiffness on the upper bound of the update rate, a larger derivative gain $k_{d}$ is adopted to satisfy the constraint $\frac{Tb+Tk_{e}k_{d}}{m+Tb+Tk_{e}k_{d}}\approx 1$. Through this parameter configuration, the stability boundary conditions for the update rate are simplified to 0<σ<0.5. This range will serve as the output domain for the ANFIS module.

### 3.3. Design of ANFIS

As proven in Section 3.2, the stability boundary conditions of the controller can be derived. However, fixed-update-rate variable impedance controllers exhibit suboptimal control performance [27], necessitating dynamic update rate adjustments to achieve enhanced control efficacy. To better align with the requirements of cleaning tasks, the dynamic update rate should adhere to the following design principles:

Initial Control Phase: During this stage, both the force-tracking error and manipulator position error of the adaptive variable impedance controller are typically large. A smaller update rate is required to mitigate force overshoot.

Steady-State Control Phase: During this stage, the force-tracking error and manipulator position error diminish significantly. To ensure a sufficient reduction in force steady-state error, a larger update rate must be employed to guarantee precision in force tracking.

Traditional fuzzy controllers can achieve dynamic parameter adjustment but typically rely on expert knowledge to manually define fuzzy rules [37,38]. It usually includes the following: setting the fuzzy subsets of the input parameters, determining the range of the domain scopes of individual parameters, designing the affiliation function of the parameters, and, finally, building a fuzzy control rule table based on the experience of experts [39]. To address the limitations of conventional fuzzy controllers, a fuzzy neural network (FNN) can be employed to automate fuzzy rule learning and enable adaptive parameter tuning. A typical FNN architecture comprises a fuzzification layer, a fuzzy rule layer, a defuzzification layer, and a fuzzy inference engine [40]. In this study, we adopt the Takagi–Sugeno model-based ANFIS as an advanced alternative. By integrating the semantic reasoning capabilities of fuzzy logic systems (FLSs) with the nonlinear learning abilities of artificial neural networks (ANNs), ANFIS achieves a smooth modulation of the controller’s update rate.

In Takagi–Sugeno fuzzy inference frameworks employing dual inputs and a single output, e.g., two inputs x and y, where x is partitioned into the fuzzy set $A_{1},A_{2},\ldots ,A_{m}$ and y is partitioned into the fuzzy set $B_{1},B_{2},\ldots ,B_{n}$, the j−th rule can usually be represented as follows [41]:

If x is $A_{p}^{j}$ and y is $B_{q}^{j}$, then $f_{j}(x,y)=\alpha_{j}x+\beta_{j}y+\gamma_{j}$.

Here, $A_{p}^{j}$ and $B_{q}^{j}$ represent the fuzzy linguistic sets corresponding to the j−th rule’s input and p≤m,q≤n; and $f_{j}$ denotes the output of the j−th rule. These parameters $p_{j},\;q_{j},\;r_{j}$ are referred to as the consequent parameters.

**Figure 4:**
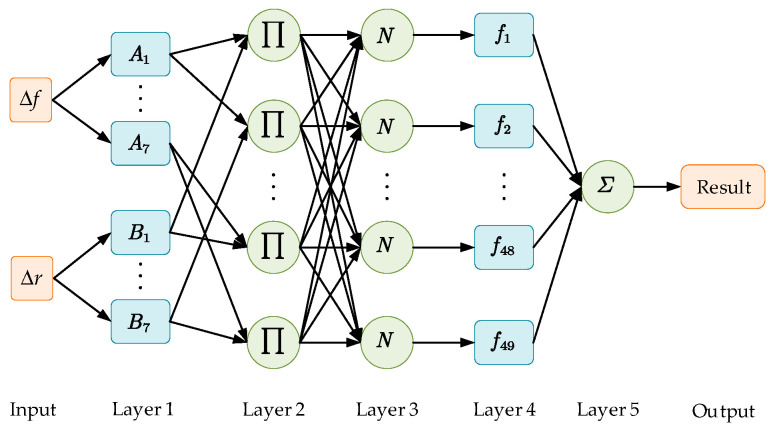
Structure of ANFIS.

In this paper, we need to dynamically calculate the update rate based on the force-tracking error and the rate of change of the force-tracking error. As described in Reference [41], the proposed ANFIS adopts a five-layer architecture, as illustrated in Figure 4. The detailed explanation of each layer is as follows:

Layer 1: This layer computes the membership degree of each input variable to its corresponding fuzzy set. It incorporates two input variables: the force-tracking error Δf and the rate of force-tracking error change Δr. The node function for each adaptive node i is defined as follows:

(29) $$\left\{\begin{array}{ll} O_{1,i}=\mu_{A_{i}}(\Delta f), & i=1,2,\ldots ,7\\ O_{1,i}=\mu_{B_{i-7}}(\Delta r), & i=8,9,\ldots ,14 \end{array}\right.$$

where $A_{j}$ and $B_{j}$ are the fuzzy linguistic labels assigned to the inputs, $O_{1,i}$ is the output of the i−th node in Layer 1, and the membership functions employ Gaussian and triangular forms.

Layer 2: The output of each node represents the activation strength of the rule. which is the product of the affiliation of the input variables, as shown in Equation (30):

(30) $$O_{2,j}=\omega_{j}=\mu_{A_{i}}(\Delta f)\cdot \mu_{B_{i}}(\Delta r),i=1,2,\ldots ,7,j=1,2,\ldots ,49$$

where j is the rule number and there are 49 rules, and $\omega_{j}$ is the activation strength of each rule.

Layer 3: This layer normalizes the triggering strength of each rule to ensure that the weights sum to 1, avoiding certain rules from over-dominating the output. The function is expressed as follows:

(31) $$O_{3,j}={\bar{\omega }}_{j}=\frac{\omega_{j}}{\sum_{j=1}^{49}\omega_{j}},j=1,2,\ldots ,49$$

where ${\bar{\omega }}_{j}$ is the weight of the normalized rule and the denominator is the sum of the activation strengths of all rules.

Layer 4: This layer calculates the output of each rule, which can be a constant or a linear function. In this work, a linear function is adopted:

(32) $$O_{4,j}={\bar{\omega }}_{j}\cdot f_{j}={\bar{\omega }}_{j}(p_{j}\Delta f+q_{j}\Delta r+r_{j}),j=1,2,\ldots ,49$$

where $\left\{p_{j},q_{j},r_{j}\right\}$ denotes the consequent parameter set of the fuzzy rules.

Layer 5: This layer aggregates the weighted outputs of all rules to produce the final crisp output:

(33) $$O_{5,j}=\sum_{j=1}^{49}O_{4,j}=\sum_{j=1}^{49}{\bar{\omega }}_{j}(p_{j}\Delta f+q_{j}\Delta r+r_{j})=\frac{\sum_{j=1}^{49}\omega_{j}f_{j}}{\sum_{j=1}^{49}\omega_{j}},\;j=1,2,\ldots ,49$$

Regarding the choice of the membership function, common membership functions include the Gaussian membership function, triangular membership function, and trapezoidal membership function. The Gaussian membership function is suitable for continuous and smooth fuzzy sets, suitable for simulating data with a clear central tendency and gradual transition, which helps to improve the smoothness of fuzzy rules; the triangular membership function is suitable for symmetric or single-peaked fuzzy sets, which has high computational efficiency, and only needs three parameters (left boundary, right boundary, and vertex) to divide the input space efficiently, which provides a good balance between the smoothness of the rules and the computational efficiency. The trapezoidal membership function is an extension of the triangular membership function, which is suitable for describing multi-peaks or flat fuzzy sets, and requires more parameters to be defined, and, at the same time, increases the complexity of the model and the computational burden. There are also the Sigmoid membership function (suitable for describing monotonically increasing or decreasing fuzzy sets), Z-shaped membership function (suitable for describing decreasing fuzzy sets), and so on. In summary, this paper chooses a compromise between fuzzy rule smoothness and computational efficiency, i.e., a combination of Gaussian and triangular membership functions.

The ANFIS employs a hybrid-learning algorithm for parameter optimization, which operates in two sequential phases: forward propagation and backward propagation. During the forward phase, the premise parameters maintain static values, whereas the consequent parameters undergo refinement through least squares optimization to minimize the output error. In the backward phase, the consequent parameters are held constant, and the premise parameters are adjusted via gradient descent to further reduce the error. To enhance the model’s generalizability, Gaussian noise with an amplitude equivalent to  of the input range is injected into the training data, while boundary constraints are enforced to ensure physical feasibility.

**Figure 5:**
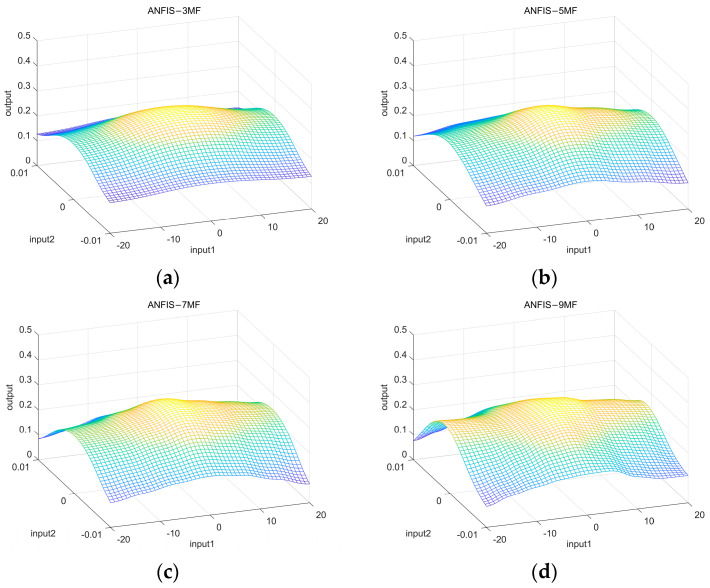
Fuzzy rule surfaces with different numbers of membership functions: (**a**) 3 membership functions; (**b**) 5 membership functions; (**c**) 7 membership functions; and (**d**) 9 membership functions.

In order to verify the reasonableness of using seven membership functions for the input variables of ANFIS, preliminary experiments are designed in this paper for verification. The experiments were set up with the number of membership functions less than 7 (e.g., 3 and 5) or more than 7 (e.g., 9) and the average absolute error of the model was measured, and the fuzzy rule surfaces with different numbers of membership functions are shown in Figure 5. The experimental results show that the mean absolute error corresponds to 0.0198, 0.0177, and 0.0156, when the number of membership functions is 3, 5, and 7, respectively. And, when the number of membership functions is 9, the average absolute error is 0.0150; the average absolute error is not significantly reduced, but the computational burden is increased. Therefore, this paper uses seven membership functions for each input variable.

## 4. Simulation Experiment

This section evaluates the force-tracking performance of the controller through simulation experiments. To closely replicate real-world cleaning task scenarios, the simulations encompass four distinct force-tracking environments, the planar surface, slope surface, sinusoidal surface, and complex curved surface, with tracking targets including both constant forces and sinusoidal forces. An ANFIS-based adaptive variable impedance controller (PID-P-ANFIS-AVIC) model was developed on the MATLAB R2023b/Simulink platform, alongside three comparative algorithms: AIC [25], AVIC [22], and PID-P-AVIC. Among them, AIC is based on a conventional impedance model with damping and stiffness coefficients adjusted online by a fixed adaptive law, while AVIC adjusts the damping coefficients online by a fixed adaptive law. Compared to PID-P-ANFIS-AVIC, PID-P-AVIC does not integrate fuzzy control or ANFIS for the adaptive adjustment of the update rate. A force sensor model is embedded in the simulation to mimic the contact force perception of an actual manipulator. The key simulation parameters were configured as follows: filtering factor h=0.007, sampling rate T=0.005 s, update rate σ=0.01, acceleration factor r=3500, and supplementary simulation variables are detailed in Table 1.

### 4.1. Force Tracking on a Plane

**Figure 6:**
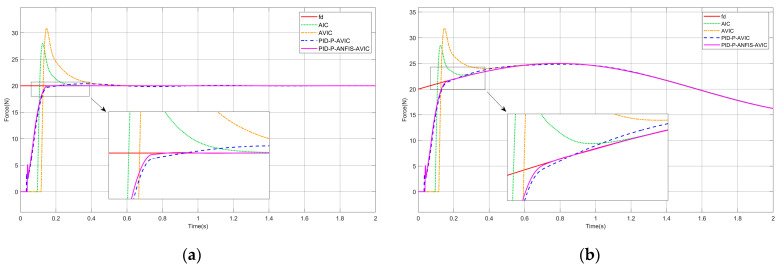
Simulation of force tracking on a plane: (**a**) constant force-tracking experiment; and (**b**) sinusoidal force-tracking experiment.

Presuming the interaction scenario constitutes a plane, the environment position is expressed as $x_{e}=const$, which satisfies ${\dot{x}}_{e}=0,{\ddot{x}}_{e}=0$. The simulation results are shown in Figure 6, and the maximum overshoot of each algorithm is given in Table 2, which can be analyzed as follows: whether tracking a constant or sinusoidal force, PID-P-ANFIS-AVIC reaches a steady state in the shortest time with little force overshoot; PID-P-AVIC also reaches a steady state very quickly with a small maximum overshoot; however, in contrast, the AIC and AVIC exhibit significantly longer settling times and substantial overshoots. Regarding force steady-state errors, all controllers (AIC, AVIC, PID-P-AVIC, and PID-P-ANFIS-AVIC) achieve zero error (0N) during constant force tracking, whereas, for sinusoidal force tracking, the force steady-state error decrease sequentially from AIC (0.15N) to PID-P-ANFIS-AVIC (0.001N), conclusively validating the PID-P-ANFIS-AVIC’s superior performance in planar force tracking for both static and dynamic profiles.

### 4.2. Force Tracking on the Slope Surface

**Figure 7:**
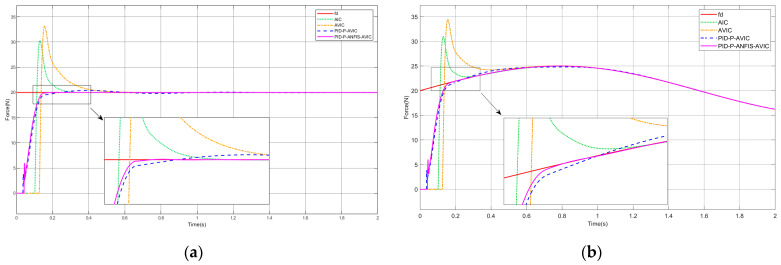
Simulation of force tracking on the slope surface: (**a**) constant force-tracking experiment; and (**b**) sinusoidal force-tracking experiment.

Presuming the interaction scenario constitutes a slope surface, the environment position satisfies ${\dot{x}}_{e}\neq 0,{\ddot{x}}_{e}=0$. The simulation results are shown in Figure 7, and the maximum overshoot of each algorithm is given in Table 3, which can be analyzed as follows: for both constant and sinusoidal force tracking, the PID-P-ANFIS-AVIC achieves the shortest settling time, demonstrates a near-zero force steady-state error, and exhibits a minimal overshoot of merely 0.03 N. In contrast to other algorithms, which exhibit a degraded performance on slope surfaces, the PID-P-ANFIS-AVIC maintains a robust force-tracking performance, underscoring its superior adaptability and precision in dynamic environments.

### 4.3. Force Tracking on the Sinusoidal Surface

**Figure 8:**
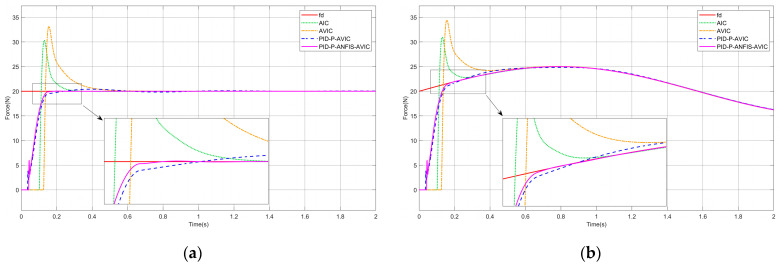
Simulation of force tracking on the sinusoidal surface: (**a**) constant force-tracking experiment; and (**b**) sinusoidal force-tracking experiment.

Assuming that the contact environment is a sinusoidal surface, the environment position satisfies ${\dot{x}}_{e}\neq 0,\;{\ddot{x}}_{e}\neq 0$. The simulation results are shown in Figure 8, and the maximum overshoot of each algorithm is given in Table 4, which can be analyzed as follows: the time required for AIC, AVIC, PID-P-AVIC, and PID-P-ANFIS-AVIC to come into contact with the environment decreases in order, the force steady-state error in tracking the constant force are all 0N, and gradually decrease when tracking a sinusoidal force. Among them, PID-P-ANFIS-AVIC has almost no force overshoot throughout the contact process, which is seen to be still very effective in force tracking on the sinusoidal plane.

### 4.4. Force Tracking on the Complex Surface

**Figure 9:**
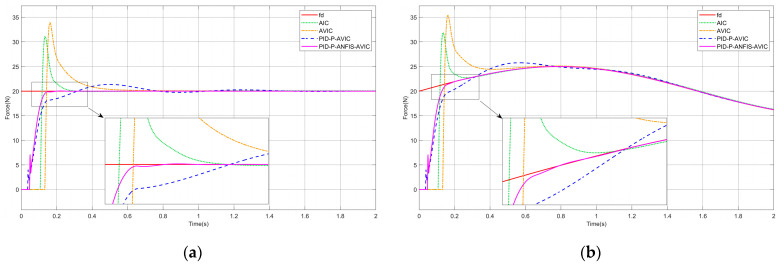
Simulation of force tracking on the complex surface: (**a**) constant force-tracking experiment; and (**b**) sinusoidal force-tracking experiment.

In practice, cleaning tasks often require a high accuracy of force control to be maintained on the complex surface. To test the force-tracking effect of PID-P-ANFIS-AVIC on complex surfaces, the simulation experiments in this section are set up. The experimental results are shown in Figure 9, and the maximum overshoot of each algorithm is given in Table 5, which can be analyzed as follows: when the environmental position becomes more complex, the tracking effectiveness of AIC, AVIC, and PID-P-AVIC all degrades, which is reflected in the fact that the force steady-state error and overshoot become larger, and the time to reach the steady state becomes longer, while the time to reach the steady state of PID-P-ANFIS-AVIC is still the shortest. And PID-P-ANFIS-AVIC still has a force steady-state error of 0 N and a maximum overshoot of 0.05 when tracking the constant force, and a force steady-state error of 0.01 N and a maximum overshoot of 0.02 N when tracking the sinusoidal force. Compared to other algorithms, PID-P-ANFIS-AVIC is able to track forces better on the complex surface.

### 4.5. Sudden Changes in Environmental Stiffness and Expected Force

To better highlight the force-tracking performance and stability of the PID-P-ANFIS-AVIC, this paper also designs the simulation of ambient stiffness mutation, constant force mutation, and sinusoidal force mutation. They are represented by Equations (34)–(36), respectively:

(34) $$k_{e}=\left\{\begin{array}{c} 10000\;(N/m),\\ 14000\;(N/m),\\ 18000\;(N/m), \end{array}\begin{array}{c} 0\leq t<0.7\\ \;0.7\leq t<1.2\\ 1.2\leq t\leq 2 \end{array}\right\}$$

(35) $$f_{d}=\left\{\begin{array}{c} 20\;(N),\\ 35\;(N),\\ 45\;(N), \end{array}\begin{array}{c} 0\leq t<0.8\\ \;0.8\leq t<1.6\\ 1.6\leq t\leq 2 \end{array}\right\}$$

(36) $$f_{d}^{\prime }=\left\{\begin{array}{c} 20+5\sin(2t)\;(N),\\ 20+10\sin(2t)\;(N),\\ 15+10\sin(2t)\;(N), \end{array}\begin{array}{c} 0\leq t<0.8\\ \;0.8\leq t<1.6\\ 1.6\leq t\leq 2 \end{array}\right\}$$

**Figure 10:**
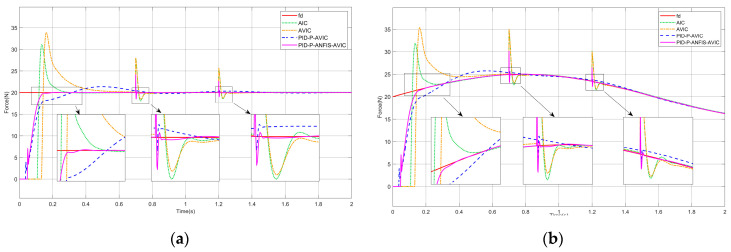
Force tracking simulation during sudden changes in environmental stiffness: (**a**) constant force-tracking experiment; and (**b**) sinusoidal force-tracking experiment.

**Figure 11:**
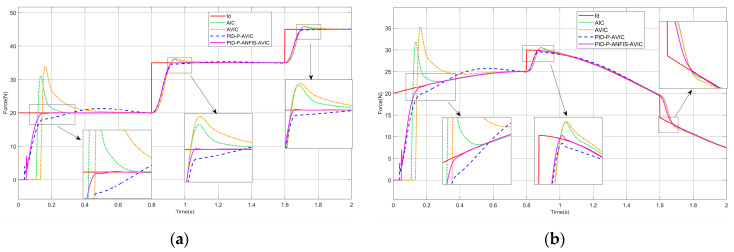
Force tracking simulation during sudden changes in expected force: (**a**) constant force-tracking experiment; and (**b**) sinusoidal force-tracking experiment.

In the simulation, we still assume that the contact environment is a complex surface, and the simulation results are shown in Figure 10 and Figure 11. Table 6 and Table 7 also give the maximum overshoot of each algorithm at each mutation. The analysis results can be seen: at the instant of a sudden change in environmental stiffness, whether tracking the constant force or sinusoidal force, the overshooting amount of each algorithm increases, among which the overshooting amount of PID-P-AVIC and PID-P-ANFIS-AVIC is relatively small, and PID-P-ANFIS-AVIC takes the shortest adjustment time, and it can continue to track the desired force very quickly. When tracking the constant force, at the instant of sudden change in desired force, AIC and AVIC will have a large amount of overshoot, PID-P-AVIC will have a small amount of overshoot, and they both need a period to continue tracking the desired force, while PID-P-ANFIS-AVIC has almost no overshoot and can continue tracking the desired force very quickly. When tracking the sinusoidal force, PID-P-ANFIS-AVIC has a small amount of overshoot and can track the desired force faster compared to PID-P-AVIC. The experimental results demonstrate that PID-P-AVIC has a better stability and force-tracking performance when the environmental stiffness and the desired force change abruptly.

### 4.6. Sensitivity Analysis of Key Parameters

**Figure 12:**
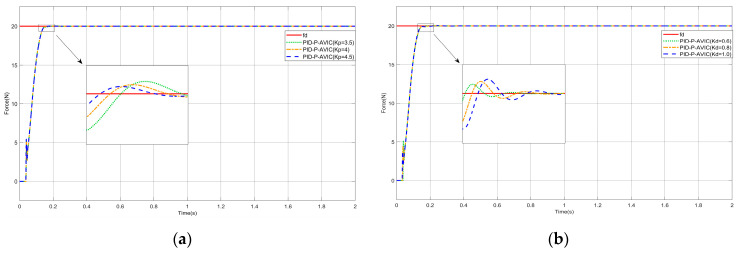
Simulation of constant force tracking on a plane: (**a**) results of the effect of changes in $k_{p}$; and (**b**) results of the impact of changes in $k_{d}$.

In order to verify the sensitivity of PID-P-ANFIS-AVIC to the variation in key parameters, this paper also designs the simulation under different $k_{p}$ and $k_{d}$. In the simulation, we assume that a constant force is tracked under the plane, and the simulation results are shown in Figure 12. Figure 12a adjusted $k_{p}$ to 3.5, 4, and 4.5, respectively, and other parameters were kept constant; Figure 12b adjusted $k_{d}$ to 0.6, 0.8, and 1, respectively, and other parameters were kept constant. From the results, it can be seen that, as $k_{p}$ increases, the response time of the system speeds up, the time to reach the steady state decreases, and the amount of overshoot decreases; and, as $k_{d}$ increases, the response time of the system slows down, the time to reach the steady state increases, and it triggers an increase in overshoot. This sensitivity analysis provides theoretical guidance and a reference basis for the adjustment of controller parameters in practical engineering, which helps us to better utilize the performance of the controller and meet the control requirements in different scenarios.

## 5. Conclusions

In this paper, an AVIC method based on ANFIS is proposed for the dynamic contact force tracking and overshoot suppression problem of the manipulator in cleaning tasks. Firstly, the contact dynamics model is constructed and combined with NTD to realize the smooth transition of the desired force and effectively reduce the contact force overshooting. In the controller design, PID control is introduced based on the traditional impedance model, and a proportional (P) control term is introduced at the parameter update rate, and then the stability conditions of the system are rigorously derived, and ANFIS is introduced to dynamically adjust the update rate to achieve the optimization of the damping and stiffness coefficients. Finally, simulation experiments are carried out in planar, slope, sinusoidal, and complex surface environments and compared with AIC, AVIC, and PID-P-AVIC, and the results show that the adaptive variable impedance controller based on ANFIS is almost free of overshooting during force tracking, and can reach the steady state faster and with less force steady-state error. Even in the case of a sudden change in stiffness or desired force, the force overshoot of this controller is very small, and it can continue to track the desired force very quickly.

The control method designed in this paper has a wide range of applicability and does not require expert knowledge to set the fuzzy rules manually, so it can be applied to other scenarios that require both position and force. While the simulation environment has comprehensively validated the adaptability of the controller in complex surfaces, the transition to a physical implementation is still critical for real-world deployment. In real robot systems, the model parameters and dynamic properties involved in manipulator kinematics and force sensors are often difficult to model accurately, and may introduce unmodeled dynamics. This can lead to deviations between the actual system response and the theoretical model predictions, thus affecting the performance of the controller. The next phase of work will focus on conducting physical experiments to validate the proposed approach. In particular, how to bridge the gap between simulation and practice on irregular surfaces. These physical experiments will provide valuable insights into the robustness and adaptability of the method in real cleaning applications.

## Figures and Tables

**Table 1:** Supplementary simulation variables.

Parameters	Value
m(kg)	1
b(N/(m/s))	120
k(N/m)	800
$k_{e}(N/m)$	10,000
η	0.1
$k_{p}$	4
$k_{i}$	0.01
$k_{d}$	2
$f_{d}(N)$	20
$f_{d}^{\prime }(N)$	20+5sin(2t)

**Table 2:** Experimental results of force tracking on a plane.

Algorithm	$\mathrm{Maximum}\;\mathrm{Overshoot}\;(f_{d})$	$\mathrm{Maximum}\;\mathrm{Overshoot}\;(f_{d}^{\prime })$
AIC	7.65 N	7.24 N
AVIC	11.24 N	10.40 N-
PID-P-AVIC	0.32 N	0.30 N
PID-P-ANFIS-AVIC	0.03 N	0.02 N

**Table 3:** Experimental results of force tracking on the slope surface.

Algorithm	$\mathrm{Maximum}\;\mathrm{Overshoot}\;(f_{d})$	$\mathrm{Maximum}\;\mathrm{Overshoot}\;(f_{d}^{\prime })$
AIC	10.05 N	9.64 N
AVIC	13.15 N	12.90 N
PID-P-AVIC	0.41 N	0.38 N
PID-P-ANFIS-AVIC	0.03 N	0.02 N

**Table 4:** Experimental results of force tracking on the sinusoidal surface.

Algorithm	$\mathrm{Maximum}\;\mathrm{Overshoot}\;(f_{d})$	$\mathrm{Maximum}\;\mathrm{Overshoot}\;(f_{d}^{\prime })$
AIC	10.25 N	9.64 N
AVIC	13.20 N	12.90 N
PID-P-AVIC	0.35 N	0.31 N
PID-P-ANFIS-AVIC	0.04 N	0.02 N

**Table 5:** Experimental results of force tracking on the complex surface.

Algorithm	$\mathrm{Maximum}\;\mathrm{Overshoot}\;(f_{d})$	$\mathrm{Maximum}\;\mathrm{Overshoot}\;(f_{d}^{\prime })$
AIC	11.20 N	10.51 N
AVIC	13.81 N	13.70 N
PID-P-AVIC	1.30 N	1.27 N
PID-P-ANFIS-AVIC	0.05 N	0.02 N

**Table 6:** Experimental results of force tracking during sudden changes in environmental stiffness.

Sudden Change	Algorithm	Maximum $\mathrm{Overshoot}\;(f_{d})$	Maximum $\mathrm{Overshoot}\;(f_{d}^{\prime })$
First mutation	AIC	7.89 N	9.84 N
	AVIC	8.07 N	10.04 N
	PID-P-AVIC	4.16 N	5.02 N
	PID-P-ANFIS-AVIC	3.72N	4.89 N
Second mutation	AIC	5.66 N	6.70 N
	AVIC	5.71 N	6.63 N
	PID-P-AVIC	2.62 N	3.07 N
	PID-P-ANFIS-AVIC	2.29 N	2.63 N

**Table 7:** Experimental results of force tracking during sudden changes in expected force.

Sudden Change	Algorithm	Maximum $\mathrm{Overshoot}\;(f_{d})$	Maximum $\mathrm{Overshoot}\;(f_{d}^{\prime })$
First mutation	AIC	0.93 N	0.74 N
	AVIC	1.25 N	0.79 N
First mutation	PID-P-AVIC	0.34 N	0.30 N
	PID-P-ANFIS-AVIC	0.03 N	0.02 N
Second mutation	AIC	0.85 N	0.20 N
	AVIC	0.92 N	0.41 N
	PID-P-AVIC	0.05 N	0.02 N
	PID-P-ANFIS-AVIC	0.03 N	0.01 N

## Data Availability

Data is contained within the article.

## Abbreviations

The following abbreviations are used in this manuscript:

AIC	Adaptive impedance control
ANFIS	Adaptive neuro-fuzzy inference system
AVIC	Adaptive variable impedance control
ANN	Artificial neural network
FLS	Fuzzy logic system
FNN	Fuzzy neural network
HFPC	Hybrid force/position control
NTD	Nonlinear tracking differentiator
PID	Proportional–integral–derivative
